# Development of a serum-free liquid medium for *Bartonella* species

**DOI:** 10.1007/s12223-016-0448-9

**Published:** 2016-02-02

**Authors:** Andreas Müller, Michael Reiter, Katrin Mantlik, Anna-Margarita Schötta, Hannes Stockinger, Gerold Stanek

**Affiliations:** Institute for Hygiene and Applied Immunology, Center for Pathophysiology Infectiology and Immunology, Medical University of Vienna, Kinderspitalgasse 15, 1090 Vienna, Austria

## Abstract

The genus *Bartonella* comprises numerous species with at least 13 species pathogenic for humans. They are fastidious, aerobic, Gram negative, and facultative intracellular bacteria which cause a variety of human and non-human diseases. This study focused on the development of a serum-free liquid medium for culture of *Bartonella* species. Some liquid media are available commercially but all of them use undefined supplements such as fetal calf serum or defibrinated sheep blood. Our intention was to create a reproducible liquid medium for *Bartonella* species that can simply be prepared. We tested several supplements that could potentially support the growth of *Bartonella* species. Slight growth improvement was achieved with glucose and sucrose. However, hemin in particular improved the growth rate. At a temperature of 37 °C, a CO_2_ concentration of 5 %, a humidified atmosphere, and the use of the supplements glucose, sucrose, and hemin, we developed a medium that does not need serum as an undefined supplement any more. In conclusion, the newly developed medium supports growth of *Bartonella* species equal to the commercially available media but with the advantage that it has a serum-free formulation. It can be prepared fast and easy and is a useful tool in studying these bacteria.

## Introduction

*Bartonella* species are distributed worldwide (Angelakis et al. 2010; Bai et al. 2013), and some of these are regarded as emerging infectious agents. They are aerobic, Gram negative, facultative intracellular bacteria that cause a variety of human and non-human diseases. Bartonellae are pleomorphic, slightly curved rods belonging to the alpha-2 subgroup of Proteobacteria. Currently, 31 *Bartonella* species and three subspecies have been described, at least 13 of these being pathogenic for humans (Kaiser et al. 2011). Recognized forms of disease are cat-scratch disease, Carrion’s disease, trench fever, bacillary angiomatosis, and endocarditis (Houpikian and Raoult 2003). The two most prominent *Bartonella* species in Europe are *Bartonella henselae*, the main causative agent of cat-scratch disease (Brenner et al. 1993; Welch et al. 1992), and *Bartonella quintana*, which causes trench or 5-day fever (Brenner et al. 1993; Munk and da Rocha-Lima 1917).

Bartonellae use a vast number of mammals as reservoirs and various arthropods such as fleas, lice, mites, and possibly also ticks as vectors (Jacomo et al. 2002; Rolain et al. 2003). Rodents form a large proportion of the identified reservoirs of *Bartonella* species. These bacteria occur in a wide variety of habitats, which results in difficult cultivation requirements. They are fastidious, slow-growing bacteria, and most of them require a humidified atmosphere with a temperature range of 30 to 37 °C and a CO_2_ saturation of 5 %.

## Materials and methods

### *Bartonella* strains

Four strains were used in cultivation experiments: *B. henselae* Houston 1 ATCC 49882 and a *B. henselae* Houston 1 ATCC 49882 modified BadA-negative strain obtained from the Biozentrum University of Basel (Dehio and Meyer 1997), RSE247; *B. henselae* Marseille, URLLY8 obtained from the Institute for Medical Microbiology and Infection Control (Riess et al. 2007); *B. quintana* NCTC 12899 and *B. vinsonii* NCTC 12905.

### Bacterial cultivation

Bartonellae were grown either on Columbia blood agar (CBA) plates with 5 % sheep blood (BioMerieux) or in an altered Schneider’s liquid medium (aSM) (Riess et al. 2008). Colony-forming units (CFU) were counted using the dilution plate technique on CBA plates. Cultures were sampled every 24 h with three dilutions in triplicate. Cultures were checked daily using streak-plating, microscopy, and PCR. Colonies picked from CBA plates were stained with CFDA-SE cell tracer kit (Invitrogen) according to the manufacturer’s instructions and observed via fluorescent microscopy (Labophot-2, Nikon) for morphology check. Strain specificity of the cultures was tested using an in-house PCR method with the primers fw-AGATGATGATCCCAAGCCTTCTGG (Knap et al. 2007) and rev-AGTCCTCCCAGGCCCACCAATT, targeting the 16 to 23 s intergenic spacer region.

The LIVE/DEAD BacLight™ Bacterial Viability Kit (Invitrogen) for cell viability determination was used according to the manufacturer’s instructions.

### Growth experiments

During a co-culture study, we observed growth of *B. henselae* in an in-house *Borrelia* liquid medium and investigated this medium in further as a potential growth medium for *Bartonella* species. For all growth experiments, a basic medium (Table 1) was used which derived from this *Borrelia* liquid medium. In order to improve the growth-promoting characteristics of the new medium, we tested several supplements which due to literature affect the growth of *Bartonella* species (Chenoweth et al. 2004; Riess et al. 2008; Sander et al. 2000). These tests included the carbohydrates mannose, galactose, glucose, fructose, and sucrose as sources of energy and other supplements such as hemin (Sigma-Aldrich). The combinations of the basic medium with the different supplements were tested by counting CFU and measuring the absorbance at 600 nm (A_600_; Spectronic 200, Thermo Scientific and Lambda 25, Perkin Elmer).

**Table 1:** Composition of the basic medium

Inorganic salts	(mg/L)
Calcium chloride anhydrous	600
Potassium chloride	1600
Potassium dihydrogen orthophosphate	500
Magnesium sulfate	3000
Sodium chloride	2500
Sodium bicarbonate	500
Disodium phosphate (Na_2_HPO_4_)	650
Other components	
Malic acid	50
Succinic acid	600
α-Ketoglutaric acid	300
Fetal calf serum	10 % (*v/v*)

### Functional analysis of *B. henselae* grown in the new medium

The host cell invasion and intracellular replication are essential aspects of the *Bartonella* life and virulence cycles. *Bartonella* adhesin A (BadA) is a major virulence factor of *B. henselae*. Therefore, we analyzed the BadA expression of *B. henselae* Houston I and Marseille in the new liquid medium in comparison to CBA. Therefore, the *B. henselae* cultures were resuspended in SDS sample buffer and heated at 95 °C for 10 min. The SDS-PAGE was performed in 12 % gels. Coomassie Blue R250 (Applichem) was used to stain the gels.

For immunoblotting, proteins were blotted onto nitrocellulose membranes (Whatman) following by blocking in 5 % skim milk powder in 25 mmol/L Tris-HCl pH 8.0, 2.7 mmol/L KCl, 137 mmol/L NaCl, and 0.05 % Tween20 (Sigma-Aldrich). The incubation time was 8 h at 4 °C. The membrane was incubated with a BadA-specific (Riess et al. 2004) or a monoclonal anti-*B. henselae* primary antibody (Abcam) for 1 h, and for detection, a horseradish peroxidase-conjugated secondary antibody was used. Visualization was performed via chemiluminescence (Pierce).

### Statistical analyses

The data were analyzed for each supplement tested using the Independent Samples *t* test. At every data point, the error bars are shown representing the standard deviation. Statistical analyses were conducted using IBM SPSS Statistics software (version 22), with results considered significant if *P* was < 0.01.

## Results

**Fig. 1:**
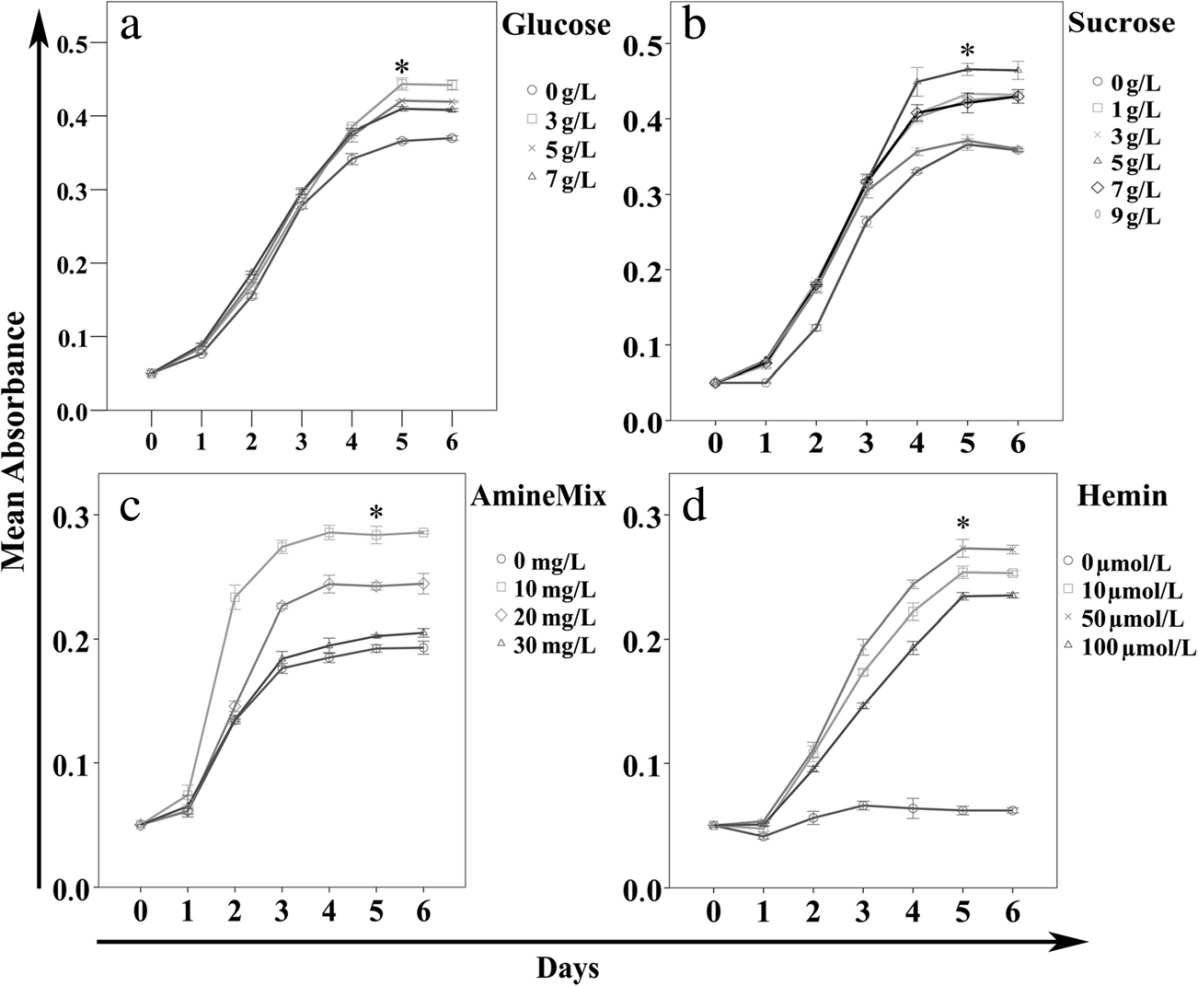
Supplement-dependent growth of *Bartonella.* The basic medium with different glucose (**a**), sucrose (**b**), amine (**c**), or hemin (**d**) concentrations was inoculated with *B. henselae* Marseille (1.0 × 10^5^ CFU/mL). The bacterial growth was determined every 24 h in triplicate by measuring the A_600_. *3 g/L glucose (a), 5 g/L sucrose (b), 10 mg/L amine (c), and 50 μmol/L hemin (d) showed a significant difference from the control value without the respective supplement (*P* < 0.01)

Compared to the basic medium, the growth of cultures was improved by the addition of glucose and sucrose (Fig. 1a, b), with the best results at 5 and 3 g/L glucose. Glucose only showed a growth improvement to a concentration of 3 g/L. Higher concentrations resulted in a growth decline. The same effect was observed with sucrose as supplement. Up to a concentration of 5 g/L sucrose had a growth improving effect. Higher concentrations resulted in a growth decline. In contrast, supplements of mannose and galactose resulted in less growth; fructose had no effect (data not shown). The N-Z-Amine-A Mix (Sigma-Aldrich) as an amino acid source gave the greatest growth improvements at 10 mg/L (Fig. 1c). With hemin (Sigma-Aldrich), the best improvements were achieved with a concentration of 50 μmol/L (Fig. 1d). Hemin, a protoporphyrin IX containing a ferric iron ion, showed the best growth improving capacities of all supplements.

**Fig. 2:**
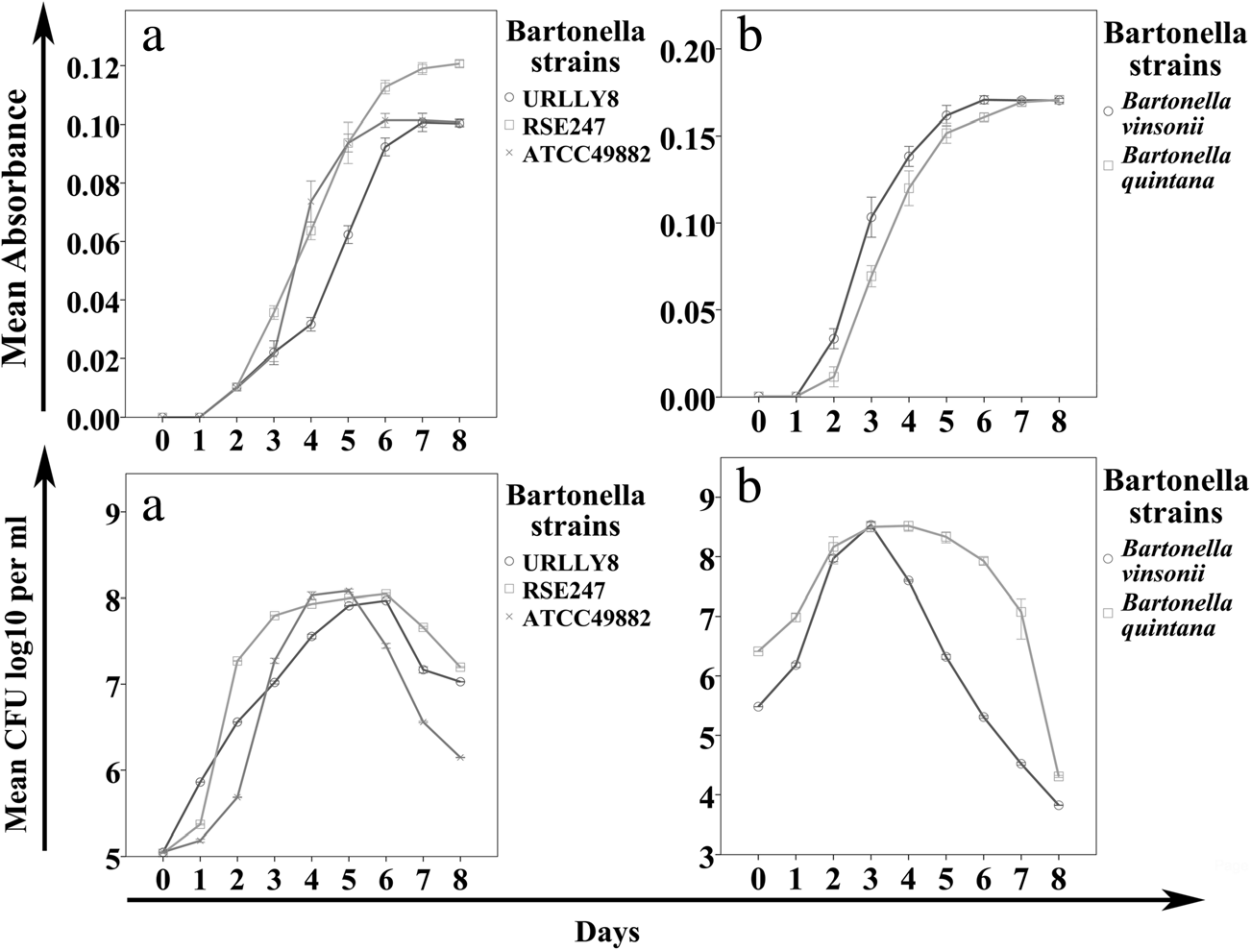
Growth curves of *Bartonella* species in the developed medium. The final medium was inoculated with different *Bartonella* strain (1.0 × 10^5^ CFU/mL), *B. henselae* Marseille URLLY8, *B. henselae* Houston 1 ATCC 49882, a modified *B. henselae* Houston 1 ATCC 49882 RSE247 (**a**), *Bartonella vinsonii* and *Bartonella quintana* (**b**). The bacterial growth was determined every 24 h in triplicate by measuring the A_600_ and by quantifying the viable bacteria (CFU/mL). Note that all strains displayed good growth in the new developed medium

The medium composition giving the best growth for bartonellae is shown in Table 2. The corresponding growth curves for *B. henselae* Houston 1 ATCC 49882, modified *B. henselae* Houston 1 ATCC 49882, and modified *B. henselae* Marseille are shown in Fig. 2a. All three strains displayed good growth to a density of 1.0 × 10^8^ CFU/mL. Furthermore, we tested *B. quintana* and *Bartonella vinsonii* with the new medium showing even better growth results (Fig. 2b).

**Table 2:** Final medium composition

Inorganic salts	(mg/L)
Calcium chloride anhydrous	600
Potassium chloride	1600
Potassium dihydrogen orthophosphate	500
Magnesium sulfate	3000
Sodium chloride	2500
Sodium bicarbonate	500
Disodium phosphate (Na_2_HPO_4_)	650
Amino acids	
N-Z-Amine A Mix	10,000
Other components	
Malic acid	50
Succinic acid	600
α-Ketoglutaric acid	300
Sucrose	5000
Glucose	3000
Hemin	50 μmol/L

**Fig. 3:**
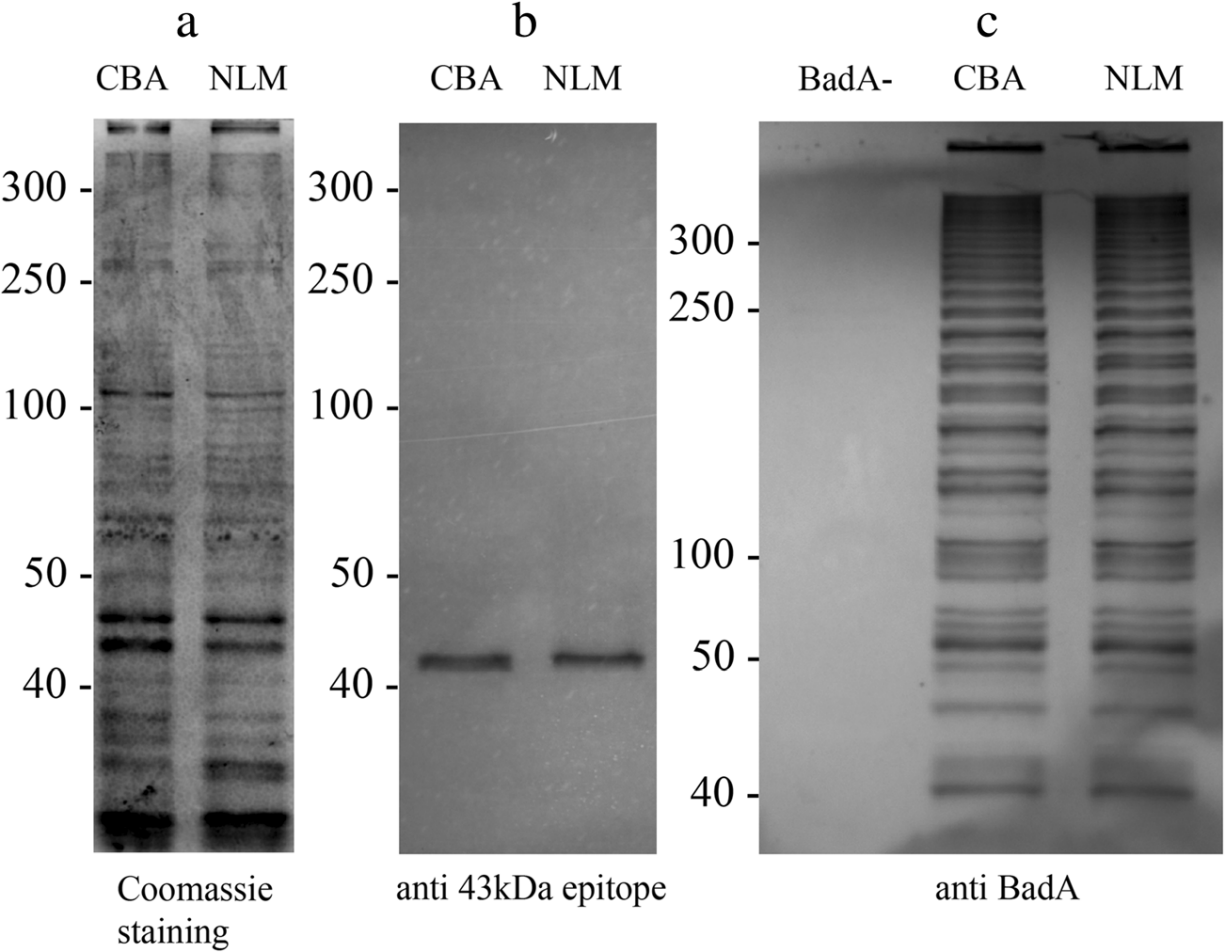
Protein composition and immunoblotting of *Bartonella henselae. Bartonella henselae* was grown either on CBA plates as well as in the new liquid medium (NLM). Protein lysates of these cultures were visualized with Coomassie blue staining (**a**) and immunoblotting was carried out with a monoclonal antibody of the 43-kDa epitope of *Bartonella henselae* (**b**). For the immunoblot analysis of BadA, the same lysates were tested, and a BadA-negative *Bartonella henselae* strain (BadA−) was used as a negative control (**c**)

The Coomassie brilliant blue staining profiles of *B. henselae* Marseille cultivated on CBA or in the new medium formulation showed no prominent differences in the total protein composition (Fig. 3a). Immunoblotting with a *B.* henselae-specific monoclonal antibody showed no differences in the expression level of the 43-kDa epitope (Fig. 3b). BadA immunoblotting resulted in the typical ladder-like pattern (Riess et al. 2008) and gave no prominent differences in the BadA pattern between *B. henselae* Marseille cultivated on CBA and in the new liquid medium (Fig. 3c).

## Discussion

We report the successful development of the first serum-free liquid medium for bartonellae. One of the most important components for a bacterial growth medium is a good carbohydrate source; however, initial tests with mannose, galactose, and fructose resulted in reduced growth or had no effect. In a genomic study, it was stated that *B. henselae* has the metabolic potential to catabolize glucose but lacks some important factors for glycolysis, such as the gene encoding phosphofructokinase, and therefore, it was suggested that elements of the Entner-Doudoroff pathway bypass this otherwise critical step of glycolysis (Canback et al. 2002). This supports our finding that fructose had no effect on the bacterial growth and that glucose facilitated growth. The same result was found for mannose, galactose, and fructose in a study with modified Schneider’s medium (Riess et al. 2008). The reason why mannose and galactose have a suppressive effect on the bacterial growth in this study is not yet understood.

The growth-improving effect of glucose is a controversial topic at present. Chenoweth et al. reported the opposite effect: *B. henselae* did not use glucose under the growth conditions they tested with an in-house medium (BBH-H) (Chenoweth et al. 2004). Thus, it is difficult to compare those results with our study where a different medium composition was used. In another study, a slight inhibition of growth was observed with 5 % (*w/v*) glucose supplementation (Riess et al. 2008), a glucose concentration more than 10 times higher than in our trials. We observed the best growth improvement with the addition of 3 g/L glucose (Fig. 1a), but increasing the glucose concentration to 5 or 7 g/L resulted in a decrease of the growth rate, which could explain the slight inhibition observed with 50 g/L (Riess et al. 2008).

The addition of sucrose was most effective at 5 g/L (Fig. 1b). A similar effect was observed in another growth study, with the difference that the sucrose concentration was 50 g/L (Riess et al. 2008).

For an amino acid source, we used the mixture N-Z-Amine-A, which contains the most important amino acids and is easy to handle for medium preparation. A concentration of 10 mg/L showed the best growth improvements (Fig. 1c). The importance of amino acids for the growth of bartonellae has been shown (Chenoweth et al.) in a study measuring depletion of free amino acids from BBH-H medium by *B. henselae* (Chenoweth et al. 2004).

Iron is an essential nutrient for viability and pathogenicity in bacteria. In the eukaryotic host, bloodstream iron is sequestered by ferritin, lactoferrin, and transferrin (Gray-Owen and Schryvers 1996; Schaible and Kaufmann 2004); thus, bartonellae, as blood-borne pathogen, needs a system for scavenging iron. Several studies have investigated the ability of bartonellae to utilize hemin, an iron-containing porphyrin found in eukaryotic blood as hemoglobin (Carroll et al. 2000; Roden et al. 2012; Sander et al. 2000; Zimmermann et al. 2003). These studies showed that *B. henselae* possesses five and *B. quintana* possesses eight outer membrane proteins known as hemin-binding proteins, and findings indicated that hemin is essential for the growth, pathogenicity, and protection of the bacteria. In the present study, a concentration of 50 μM hemin resulted in the best growth rate (Fig. 1d). A similar observation was made by Sander et al. (Sander et al. 2000).

The final medium composition including all growth-facilitating supplements (Table 2) was tested with the four *Bartonella* species used in this study (Fig. 2a, b). The growth yield for these species was between 1 and 5 × 10^8^ CFU/mL with a corresponding A_600_ between 0.1 and 0.17. In comparison with currently used liquid media for bartonellae, the maximum cell concentration was reduced by 0.5 log, but this did not affect the culture reaching the stationary phase. As in other studies, the cultures reached their stationary-phase after 6 to 7 days (Lynch et al. 2011; Riess et al. 2008).

The immunoblotting analysis of *B. henselae* Marseille grown in the new liquid medium showed the typical ladder-like band-pattern of BadA, a major pathogenicity factor of this *Bartonella* species. No differences were observed between the cultivation of *B. henselae* Marseille on CBA or in the new liquid medium (Fig. 3c). The protein composition did not show any prominent differences, and the immunoblotting with a *B. henselae*-specific monoclonal antibody revealed the same expression levels of the 43-kDa epitope of *B. henselae* (Fig. 3a, b).

To our knowledge, this is the first study that has developed a serum-free liquid medium for *Bartonella*. This reproducible medium can be prepared fast and easy and supports the growth of several *Bartonella* species. No changes of the protein expression pattern, including BadA, for *B. henselae* could have been observed. Therefore, the new liquid medium appears to be a useful tool in studying these bacteria of which some belong to the agents of emerging infectious diseases, distributed worldwide.
